# Haplotype‐Based Analysis of *OCA2* Variants in Oculocutaneous Albinism

**DOI:** 10.1111/pcmr.70085

**Published:** 2026-03-29

**Authors:** Meredith F. Gillis, Madeleine R. Ames, Linnea Lundh, Valer Gotea, Laura Elnitski, Frank Donovan, NISC Comparative Sequencing Program, Adebowale Adeyemo, Charles Rotimi, Brian Brooks, Wadih Zein, William Gahl, William S. Oetting, David R. Adams, Stacie K. Loftus

**Affiliations:** ^1^ Human Biochemical Genetics Section National Human Genome Research Institute, National Institutes of Health Bethesda Maryland USA; ^2^ Department of Cell Biology and Physiology University of North Carolina at Chapel Hill Chapel Hill North Carolina USA; ^3^ Translational and Functional Genomics Branch National Human Genome Research Institute, National Institutes of Health Bethesda Maryland USA; ^4^ Cancer Genomics Unit National Human Genome Research Institute, National Institutes of Health Bethesda Maryland USA; ^5^ NIH Intramural Sequencing Center National Human Genome Research Institute, National Institutes of Health Bethesda Maryland USA; ^6^ Center for Research in Genomics and Global Heath National Human Genome Research Institute, National Institutes of Health Bethesda Maryland USA; ^7^ Opthalmic Genetics and Visual Function Branch National Eye Institute, National Institutes of Health Bethesda Maryland USA; ^8^ Opthalmic Clinical Genetics Section National Eye Institute, National Institutes of Health Bethesda Maryland USA; ^9^ Department of Experimental and Clinical Pharmacology University of Minnesota Minneapolis Minnesota USA; ^10^ Office of the Clinical Director National Human Genome Research Institute, National Institutes of Health Bethesda Maryland USA

**Keywords:** GWAS, haplotype, *OCA2*, oculocutaneous albinism

## Abstract

OCA2, a melanosome transmembrane spanning protein, functions to regulate melanosomal pH, optimizing production of melanin pigment. *OCA2* is one of eight non‐syndromic autosomal recessive oculocutaneous albinism (OCA) loci and is the second most common cause of OCA worldwide. Genome wide association studies (GWAS) have identified *OCA2* coding and regulatory variants linked to common skin and eye color pigment variation, skin cancer susceptibility, and retinal pigment epithelium tissue metrics. Within a cohort of 106 *OCA2* probands with two biallelic *OCA2* variants, a total of 74 distinct *OCA2* rare variants were identified (11 large structural, 17 small indel/frameshift, 12 splice site, and 34 missense coding variants). Phase‐validated haplotypes, comprised of both *OCA2* common pigmentation trait GWAS alleles and rare variants, were obtained for 95/106 probands. In total, 41 distinct multi‐allele *OCA2* haplotypes were identified with 27 haplotypes containing either rs1800404‐A and/or rs12913832‐G alleles, each of which is known to reduce correct isoform splicing or gene expression by ~20%. These results find that common GWAS alleles with known *OCA2* functional impact are present on haplotypes with variants of unknown significance in *OCA2* probands and highlight the need for haplotype‐based analysis at the *OCA2* locus in addition to individual variant pathogenic assessment.

## Research Highlights

This work evaluates the pathogenicity of rare *OCA2* variants from 106 OCA type 2 individuals. The identified pigmentation trait associated, multivariant haplotypes have implications in understanding the phenotypic diversity associated with OCA type 2.

## Graphical abstract

This study defines multi‐allele haplotypes, comprised of rare disease variants combined with common eQTL, sQTL and GWAS variants, for oculocutaneous albinism type 2 (*OCA2*). These haplotypes have implications for variant pathogenicity assessments and phenotypic variability.

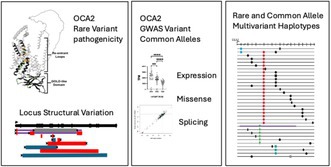

## 1 Introduction

Oculocutaneous albinism (OCA) is an autosomal recessive disorder characterized by a reduction of pigment in the hair, skin, and eyes. Hypopigmentation of the skin leads to an increased risk of UV‐induced skin cancers, while in the eye, hypopigmentation leads to poor visual acuity primarily due to foveal hypoplasia, optic nerve misrouting, and elevated rates of refractive errors. OCA is both phenotypically and genetically heterogeneous, with 8 types of non‐syndromic albinism identified by biallelic variants at 8 distinct loci: *TYR* (OCA types 1A and 1B), *OCA2* (OCA type 2), *TYRP1* (OCA type 3), *SLC45A2* (OCA type 4), *SLC24A5* (OCA type 6), *LRMDA* (OCA type 7), *DCT* (OCA type 8), and a region of 4q24 in a single consanguineous family (OCA type 5) (Thomas et al. 1993). Three loci (*TYR, TYRP1*, and *DCT*) encode melanosomal enzymes involved in melanin synthesis and two loci (*OCA2* and *SLC45A2*) modulate melanosome vesicular pH. Overall, 15%–30% of individuals with OCA who undergo molecular testing remain without a definitive molecular diagnosis (Gronskov et al. 2009; Kruijt et al. 2018; Lasseaux et al. 2018). This value includes many individuals without any indication of causative mutations in pigmentation genes and others with only a single definitive pathogenic allele detected. The world‐wide prevalence of OCA, based solely on phenotypic presentation, is difficult to determine as rates vary drastically among geographically distinct populations and may be influenced by local psychosocial and cultural differences (Kromberg et al. 2023).

The most recent estimate of OCA prevalence in European countries is ~1/13,000 with the majority of molecular diagnoses attributed to variants in genes *TYR* and *OCA2* (Kromberg and Kerr 2022). The average prevalence of OCA type 2 among subsets of African populations is estimated to be ~1/4000 with rates as high as 1/1000 in some population isolates of Sub‐Saharan Africa (Kromberg and Kerr 2022; Lund 1996). This increased prevalence is attributed to an *OCA2* founder allele (2.7 kb del) that deletes all of exon 7 and the surrounding intronic sequence (Durham‐Pierre et al. 1996; Puri et al. 1997; Stevens et al. 1997). Other frequently identified alleles include a 122.5 kb deletion prevalent among individuals with Navajo ancestry (Yi et al. 2003), missense variant c.1327G>A (p.Val433Ile) (Lasseaux et al. 2018; Loftus et al. 2021) and a complex structural variant (CxSV) comprised of an inverted duplication/deletion event spanning introns 2 to 19 (Lasseaux et al. 2018; Loftus et al. 2021; Lund 1996; Morice‐Picard et al. 2014). However, many patients present with rare variants of unknown significance (VUS) in *OCA2* and other OCA‐related genes. The combined genetic and phenotypic heterogeneity present in individuals with OCA can be confounding factors in providing definitive biallelic molecular diagnoses.

*OCA2* encodes P protein (OCA2), a transmembrane spanning protein putatively identified as a melanosomal membrane channel. While the primary sequence and computational structural modeling of OCA2 suggest similarity to the SLC13 Na^+^/dicarboxylate transporter family (Mesdaghi et al. 2023), electrophysiological studies have found that OCA2 functions as a chloride‐selective anion channel on the melanosome (Bellono et al. 2014). OCA2 neutralizes vesicular pH, thus permitting the enzymes TYR and TYRP1 to produce melanin pigment. Additional evidence suggests a potential role of OCA2 in stabilizing TYR and TYRP1 in the endoplasmic reticulum to effect proper folding and trafficking of TYR (Lamoreux et al. 1995; Manga and Orlow 2001).

Genome wide association studies (GWAS) have found that many variants located within OCA gene loci are also correlated with the broad spectrum of human hair, skin and eye pigment variation and ocular related traits commonly observed across many different populations (Cerezo et al. 2025). At the *TYR* locus, common allele frequency GWAS‐identified variants are present in cis‐orientation on a single rare haplotype, *TYR*:[c.‐301C;c.575C>A;p.Ser192Tyr;p.Arg402Gln], and found to contribute to OCA type 1B pathology (Gronskov et al. 2019; Lin et al. 2022; Loftus et al. 2023; Sergouniotis et al. 2023). These results indicate that the modulation of gene expression resulting from the combinatorial impact of a multi‐allele haplotype produces the overall OCA phenotype. This highlights that a comprehensive diagnostic approach for albinism should include full characterization of the phase orientation of variants detected within all OCA gene loci rather than an isolated focus on rare variants.

At the *OCA2*‐*HERC2* locus, multiple common GWAS variants are correlated with OCA phenotype‐related traits including skin color (Adhikari et al. 2019; Crawford et al. 2017; Farre et al. 2023; Galvan‐Femenia et al. 2018), hair color (Farre et al. 2023; Lin et al. 2015; Morgan et al. 2018; Sulem et al. 2007), susceptibility to skin cancers (Farre et al. 2023; Landi et al. 2020), eye color (Simcoe et al. 2021; Sulem et al. 2007), and retinal morphology (Currant et al. 2021; Jackson et al. 2025). *OCA2/HERC2* locus GWAS variants include missense coding variant c.1184G>A (p.Arg419Gln) [rs1800407], synonymous variant c.1065G>A (p.Ala355=) [rs1800404], and distal regulatory allele rs12913832G>A. Rs1800404‐A has also been associated with a proportional 20% reduction in overall correct *OCA2* transcript splicing per variant allele, creating an alternative splicing transcript that skips exon 10 (NM_001300984). Enhancer variant rs12913832 resides −21 kb upstream of the *OCA2* transcriptional start site (TSS) in intron 86 of the *HERC2* locus. This regulatory element has been found to interact with the OCA2 promoter, and rs12913832‐G results in an overall reduction in *OCA2* expression levels (Visser et al. 2012).

Common variants tend to be dismissed in rare disease diagnostic tests, since individual common variants have homozygous genotype frequencies in normally pigmented populations that are inconsistent with a capacity to cause the disease in isolation. However, there has been limited analysis of the frequency of multivariant haplotypes consisting of rare variant/common variant combinations or multiple *OCA2* rare variants. We present a detailed interrogation of the extended haplotype structures for chromosomes carried by individuals in an OCA2 patient cohort comprising 106 individuals. This analysis provides a more complete understanding of the underlying genetic architecture for common pigmentation trait associated alleles and identifies recurring OCA2 haplotypes associated with disease.

## 2 Materials and Methods

### 2.1 Human Subjects

DNA was obtained from individuals seen either under the NIH Clinical Center Natural History Study (IRB approved study 2009‐HG‐0035), clinical study protocol 76‐HG‐0238, or were part of a deidentified cohort of DNA samples from individuals diagnosed with OCA under IRB‐950M10178 assembled by Dr. Richard King and Dr. William Oetting at the University of Minnesota. This study conforms to the recognized standard in the US Federal Policy for the Protection of Human Subjects. Partial genetic information was previously reported for individuals 2465 (Frenk and Calame 1977); 2461, 2946, and 2622 (King et al. 2003); 1211, 1218, 1232, 2091, 2457, 2781, 3111, and 3262 (Loftus et al. 2021); and 1238, 1252, 2875, and 3064 (Loftus et al. 2023).

### 2.2 Sequencing and Read Alignment

Custom capture sequence (CCS)‐based analysis to identify *OCA2* cohort probands was performed as previously described (Loftus et al. 2021) with chromosome coordinates for identified variants lifted over to GRCh38. Individuals 1180 and 1220 were screened by PCR amplification with *OCA2* primers followed by Sanger sequencing (Table S1).

### 2.3 Variant Functional Interpretation

Assessment of OCA variants was performed using Annovar‐based variant annotation as previously described (Loftus et al. 2021). *OCA2* variants were identified relative to GenBank:NM_000275.3 using Varsome, Alumut, and ClinGen databases. American College of Medical Genetics and Genomics (ACMG) criteria were used to define pathogenicity (Richards et al. 2015). Correlation between rs12913832 genotypes and *OCA2* expression levels was obtained from high‐depth primary melanocyte RNA‐seq sequence data (Zhang et al. 2018).

### 2.4 SNP Genotyping and Copy Number Variant (CNV) Detection

Samples were genotyped using the InfiniumExome‐24v1.1 BeadChip (Illumina Inc.), and BAM files were manually evaluated using Integrative Genomics Viewer (IGV, Broad Institute) to identify structural rearrangements as previously described (Loftus et al. 2023). All junctions were confirmed by de novo realignment of paired‐reads in Sequencher (Gene Codes). Copy number variant assessment for exon 7 and exon 14 deletions was obtained using average LRR Ratios (LRR) for exonic SNPs from genome‐wide gnomAD SNP genotyping data in comparison to LRR control samples.

### 2.5 Allelic Phase

Phase was established directly from paired‐end sequence reads and segregation of variants in family trios where possible. TaqMan genotyping (Thermofisher) was performed for variants rs121918166 and rs1800404. All other alleles were evaluated by genomic PCR and Sanger sequencing (Psomagen) (Table S1). Population haplotype frequencies were calculated using LDHap and GRCh38–High coverage 1000 Genomes Project data (Machiela and Chanock 2015).

### 2.6 Splice Predictions and Protein Modeling

Multiple predictive splice programs were used to assess the potential impact of a variant on *OCA2* splicing. The program SPANR (Xiong et al. 2015) was trained on RNA‐seq data from 16 tissues from the Illumina Human Body Map 2.0 Project and can be used for single nucleotide variants located within 300 bp of annotated exons. For variants located outside the consensus splice site (GT for 5′ and AG for 3′ splice sites, respectively), SplicePort (Dogan et al. 2007) was utilized. SplicePort uses 80 bp on each side of the consensus dinucleotide to score a given splice site(SS); for *OCA2* exon 10, this corresponded to the 3′SS region chr15:27990568–27990729 and the 5′SS region chr15:27990494–27990655. For cis‐haplotype variants within 80 bp of a splice site, we calculated their compounded impact as the difference between the splice site score with both variants present from the reference sequence. For variants affecting more than one splice site, only the impact on the strongest splice site was recorded. MaxEntScan (Shamsani et al. 2019), which models 9 bp around the 5′SS and 23 bp around the 3′SS, and SpliceAI‐lookup (BroadInstitute 2025) were employed due to their widespread application in splice‐site scoring. Mol* Viewer was used to model the AlphaFold‐predicted structure of OCA2 (AF‐Q04671‐F1). The two‐dimensional model of OCA2 (Mercier et al. 2025; Mesdaghi et al. 2023) was created with BioRender. AlphaMissense scores were obtained from Tordai et al. (2024).

## 3 Results

### 3.1 Spectrum of Pathogenic Variants

**FIGURE 1:**
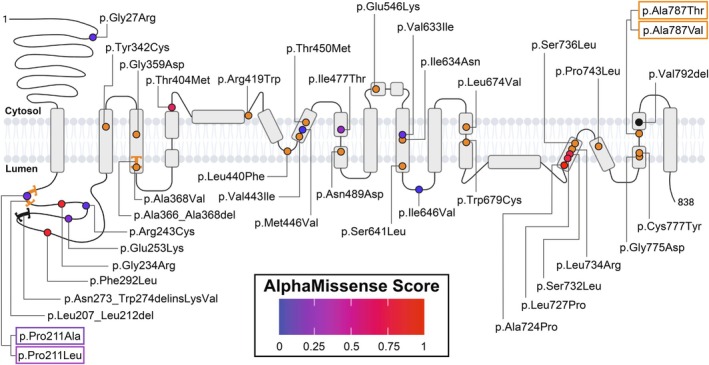
Location of cohort variants within a 2‐dimensional model of OCA2 protein structure. *OCA2* missense variants classified P/LP via ACMG criteria are colored orange. Missense *OCA2* VUS are colored according to their AlphaMissense score with 0 = likely benign and 1 = likely pathogenic. *OCA2* deletions classified as VUS (p.Asn273_Trp274delinsLysVal and p.Val792del) are black. Brackets indicate deletion and deletion–insertion variants that span multiple amino acids. For missense variants that affect the same amino acid position, classification is distinguished by the color of the box around the coding mutation label.

Our cohort includes OCA probands carrying two or more *OCA2* variants, each with a MAF < 0.01. Our analysis identified 74 distinct *OCA2* variant alleles in 106 probands (Table 1, Table S2). Alleles included 11 large structural variants (SVs) (Table S3), 17 small indel/frameshift/premature stop variants, 34 missense single‐nucleotide variants (SNVs) (Figure 1), and 12 predicted splice variants. Systematic application of ACMG criteria classified 76% of variant alleles as pathogenic or likely pathogenic (P/LP) and 24% as VUS (Table S4). A total of 20 variant alleles were not previously reported in ClinVar or found in the literature, and of these, 13 are P/LP, and the remaining are VUS. The most frequent individual SNVs identified in our cohort were c.1327G>A (p.Val443Ile) (*n* = 37), c.1465A>G (p.Asn489Asp) (*n* = 13), c.2228C>T (p.Pro743Leu) (*n* = 11), and c.1320G>C (p.Leu440Phe) (*n* = 8).

**TABLE 1:** OCA2 cohort identified variants.

ID	M/F	OCA2 variant 1	Pat	OCA2 variant 2	Mat	Phase method
*Two P/LP alleles*						
3500	F	g.28017719_28020678delinsTTT		g.28017719_28020678delinsTTT		CCS
1222	M	c.2228C>T		c.1327G>A		ND
1211	F	c.1901T>A	Pat	143 kb/184 kb CxSV	Mat	Trios
1180	F	g.28017719_28020678delinsTTT		g.28017719_28020678delinsTTT		ND
1148	M	c.1503+5G>A	Pat	c.163del	Mat	Trios
1238	M	c.1327G>A	Pat	c.1116+5G>A	Mat	Trios
1002	M	c.1503+5G>A		c.1327G>A		ND
1093	F	c.2310 T>G		c.1465A>G		ND
1218	F	c.1025A>G	Pat	143 kb/184 kb CxSV	Mat	Trios
1232	M	143 kb/184 kb CxSV	Pat	c.1327G>A	Mat	Trios
1252	F	c.79G>A; c.1320G>C	Pat	c.79G>A; c.1320G>C	Mat	CCS
1261	M	g.28017719_28020678delinsTTT		g.28017719_28020678delinsTTT		CCS
1122	F	c.1327G>A		c.1327G>A		CCS
1078	F	c.274del	Pat	c.1327G>A	Mat	Trios
2035	F	g.27837375_28079574dup		c.1327G>A	Mat	Mat
2066	F	c.2207C>T		c.2201 T>G		CCS
2091	M	143 kb inverted dup CxSV	Pat	c.1327G>A	Mat	Trios
2105	F	c.1290 T>A	Pat	c.1327G>A	Mat	CCS
2112	M	c.1327G>A		g.28017719_28020678delinsTTT		ND
2163	F	g.28017719_28020678delinsTTT		g.28017719_28020678delinsTTT		CCS
2180	M	c.1327G>A		c.2228C>T		ND
2189	F	c.79G>A; c.1320G>C	Pat	c.2330G>A	Mat	Trios
2208	F	c.2228C>T		g.27965350_27973636delinsACACAACTTCATTGATAATGGCCTCTATTTA		ND
2280	M	c.1465A>G		c.1327G>A		ND
2282	M	c.1327G>A	Pat	c.2228C>T	Mat	Trios
2379	F	c.1103C>T		c.1103C>T		CCS
2415	F	c.1327G>A		c.2433‐22,889 T>A		ND
2418	F	c.1465A>G		c.1465A>G		CCS
2422	F	c.493C>T		g.27878517_28001141del		ND
2425	M	c.2228C>T		c.1327G>A		ND
2435	M	c.1327G>A	Pat	c.79G>A; c.1320G>C	Mat	CCS
2451	M	c.1465A>G		c.1327G>A		ND
2456	F	c.1103C>T		g.28017719_28020678delinsTTT		ND
2457	M	c.1327G>A		143 kb/184 kb CxSV		
2461	F	c.1465A>G	Pat	c.2037G>C	Mat	Trios
2465	M	c.1255C>T		g.(23975851_24027348)_(28442183_28445507)del		CCS
2552	M	c.1103C>T		c.1349C>T		ND
2557	F	c.1076G>A		c.1076G>A		CCS
2587	F	c.1327G>A		c.1327G>A		CCS
2593	M	c.1465A>G	Pat	c.1327G>A	Mat	Trios
2612	M	g.28017719_28020678delinsTTT	Pat	c.1327G>A	Mat	Trios
2622	F	c.1327G>A	Pat	g.28017719_28020678delinsTTT	Mat	ND
2637	F	c.1365‐1G>C		c.163dup		Trios
2669	F	c.928C>T		c.928C>T		CCS
2682	M	c.79G>A; c.1320G>C		c.1327G>A		CCS
2766	M	c.619_636del	Pat	c.1327G>A	Mat	Trios
2781	M	c.1327G>A	Pat	143 kb/184 kb CxSV	Mat	Trios
2847	F	c.1327G>A	Pat	c.1465A>G	Mat	Trios
2854	M	c.1349C>T		g.28017719_28020678delinsTTT		ND
2875	M	c.2228C>T	Pat	c.2020C>G	Mat	Trios
2897	M	c.401G>A		c.401G>A		CCS
2938	M	g.27878517_28001141del		g.27878517_28001141del		CCS
2946	M	c.2228C>T	Pat	c.1327G>A	Mat	Trios
2960	F	c.1182+1G>A		g.27844884_28020885del		CCS
2976	M	c.1465A>G		c.1327G>A		ND
3005	F	c.1327G>A	Pat	c.2228C>T	Mat	Trios
3029	F	g.28017719_28020678delinsTTT		c.1503+5G>A		ND
3030	F	c.1503+5G>A		g.28017719_28020678delinsTTT		ND
3032	M	c.2140‐2A>G		g.28017719_28020678delinsTTT		ND
3034	F	c.1327G>A		c.612G>A		ND
3059	F	g.27844884_28020885del		g.[27975445_27984368dup;27984368_27984369insTTAACA]		CCS
3111	F	143 kb/184 kb CxSV	Pat	c.1327G>A	Mat	Trios
3112	M	c.1045_1046del	Pat	c.1922C>T	Mat	Trios
3117	F	c.228‐2A>G		g.28017719_28020678delinsTTT		ND
3139	F	c.2244+2T>G	Pat	c.574‐1G>A	Mat	Trios
3146	F	g.28017719_28020678delinsTTT		g.27973488_28073270dup		CCS
3188	F	c.79G>A; c.1320G>C		c.1327G>A		CCS
3192	F	c.1327G>A	Pat	c.1465A>G	Mat	Trios
3207	M	c.1327G>A		g.28017719_28020678delinsTTT		ND
3262	M	c.79G>A; c.1320G>C		143 kb/184 kb CxSV		ND
3264	F	c.406C>T		c.1255C>T		ND
3273	F	c.1349C>T	Pat	c.1636G>A	Mat	Trios
3285	M	c.2228C>T		c.2228C>T		CCS
3289	M	g.27979571_27984604del		g.27979571_27984604del		CCS
3290	F	g.27979571_27984604del		g.28017719_28020678delinsTTT		ND
3291	F	g.28017719_28020678delinsTTT		g.27979571_27984604del		ND
3293	F	g.28017719_28020678delinsTTT		g.28017719_28020678delinsTTT		CCS
3294	F	g.28017719_28020678delinsTTT		c.2360C>T		ND
3295	M	g.28017719_28020678delinsTTT		g.28017719_28020678delinsTTT		CCS
3296	M	g.27979571_27984604del		g.28017719_28020678delinsTTT		ND
*One LP or P allele and one VUS*						
3501	M	c.2324G>A		c.1239+5G>C		ND
1220	F	c.1897G>A		c.1255C>T	Mat	Mat
1225	M	c.1465A>G	Pat	c.1336A>G	Mat	Trios
2055	M	c.1327G>A	Pat	c.632C>T	Mat	Trios
2244	F	c.1095_1103del		c.1430T>C	Mat	ND
2546	M	c.1465A>G		c.2180T>C		ND
2556	M	c.2228C>T		c.1239+5G>C		ND
2666	F	g.28017719_28020678delinsTTT		c.632C>T		ND
2726	M	c.1951+1G>A		c.874 T>C	Mat	Trios
2784	M	g.28017719_28020678delinsTTT	Pat	c.1239+5G>C	Mat	Trios
2837	F	c.819_822delinsGGTC	Pat	c.2359G>A	Mat	Trios
2856	M	c.414C>A	Pat	c.1936A>G	Mat	Trios
2912	M	c.700G>A	Pat	c.1465A>G	Mat	Trios
2981	F	c.2370_2375delinsCGT		c.79G>A; c.1320G>C	Mat	Mat
3064	F	c.1555delG	Pat	c.632C>T	Mat	Trios
3258	M	c.2201T>G		c.631C>G		ND
3288	F	g.27979571_27984604del		c.2170G>C		ND
3292	F	g.27979571_27984604del		c.2170G>C		ND
*Two VUS alleles*						
3502	M	c.1239+5G>C		c.1239+5G>C		CCS
1209	M	c.1211C>T		c.2245‐3_‐2del	Mat	ND
1064	M	c.819_822delinsGGTC	Pat	c.2195C>T	Mat	Trios
1275	F	c.1239+5G>C		c.1239+5G>C		CCS
2431	F	c.757G>A	Pat	c.727C>T	Mat	CCS
2720	F	c.819_822delinsGGTC	Pat	c.874T>C	Mat	CCS
2795	F	c.819_822delinsGGTC		c.819_822delinsGGTC		CCS
2820	F	c.874T>C		c.874T>C		CCS

*Note:* 143 kb/184kb CxSV: NC_000015.10:g.[25834403‐25836784delins] [CCTGGTTGTAGGTCTAACCTGGTTAGAATCT; 25640598_25783343inv;C]; [25617295_25801161del]; 143kb inverted dup CxSV, NC_000015.10:g.[25834403‐25836784delins] [CCTGGTTGTAGGTCTAACCTGGTTAGAATCT;25640598_25783343inv; C].

Abbreviations: CCS, custom capture sequence; F, female; M, male; Mat, maternal inherited allele; ND, not determined; Pat, paternal inherited allele.

### 3.2 AlphaMissense and AlphaFold Variant Evaluation

**FIGURE 2:**
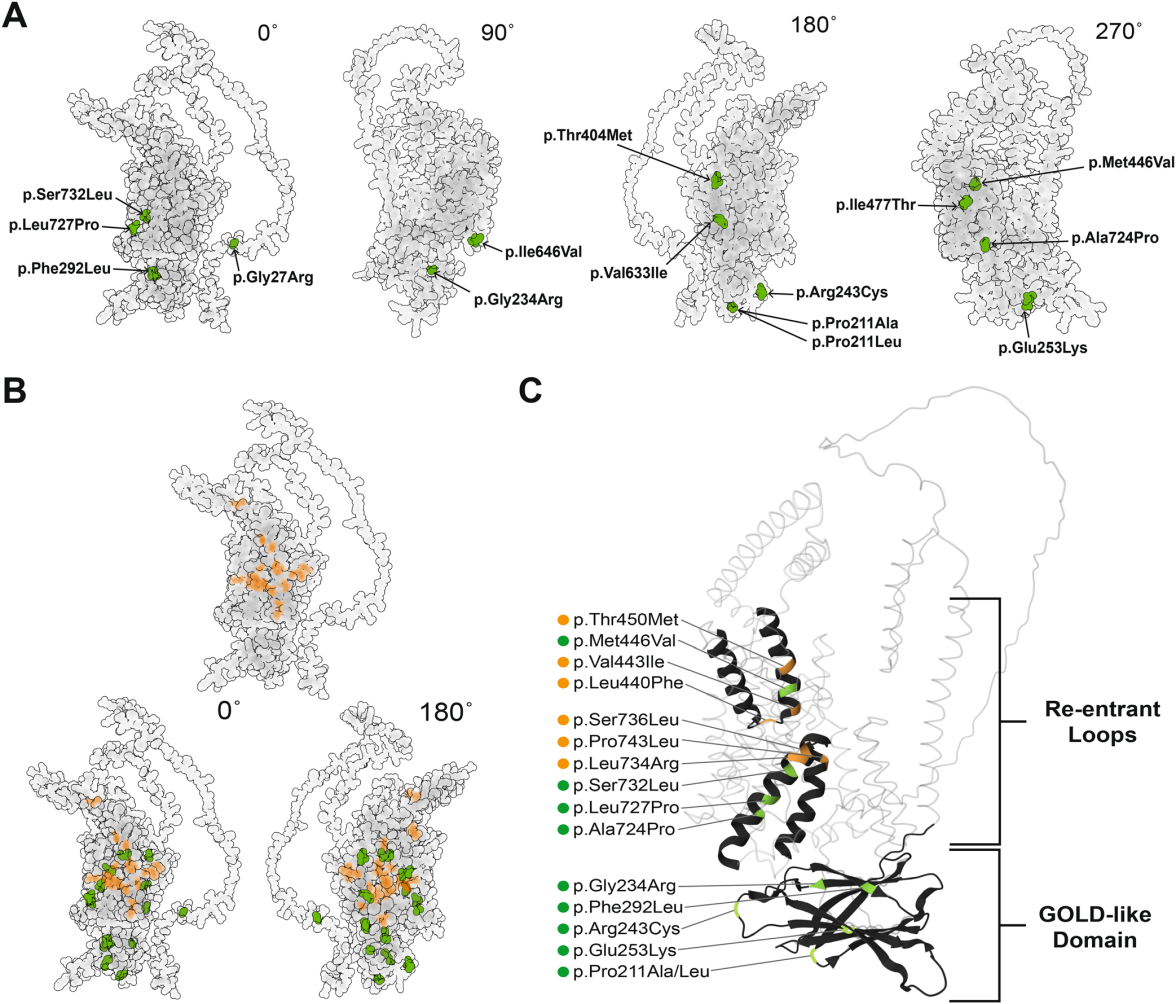
Location of missense OCA2 cohort variants in a 3‐dimensional model of OCA2 protein structure. (A) A 3D model of OCA2 (AF‐Q04671‐F1) is rotated at 0°, 90°, 180°, and 270°. Missense OCA2 VUS are labeled in green on the face of the protein where they are most visible. (B) P/LP missense OCA2 variants are colored orange on the front view of OCA2. Missense VUS and P/LP variants are colored concurrently on front and back views of the 3D OCA2 model. (C) Missense P/LP variants and VUS located in the re‐entrant loops and the GOLD‐like domain of OCA2 predicted by Mesdaghi et al. are highlighted.

Missense SNVs in our cohort were visualized on the predicted OCA2 3‐D structure using AlphaFold (Figure 2). Missense P/LP variants primarily localize to central structures that span the melanosomal membrane and include the elevator helix structures predicted by Mesdaghi et al. (2023), whereas missense VUS tend to be located at the periphery of the core membrane spanning region (Figure 2B,C). All missense SNVs were assessed by AlphaMissense and their scores were evaluated for concordance with ACMG pathogenicity criteria. Of the 20 ACMG‐defined P/LP missense SNVs, 14 were predicted to be LP by AlphaMissense (Figure 1, Table S4). Of the six non‐concordant LP assigned alleles, AlphaMissense predicted two as likely benign, c.1255C>T (p.Arg419Trp) and c.2020C>G (p.Leu674Val), and four as ambiguous, c.1103C>T (p.Ala368Val), c.1327G>A (p.Val443Ile), c.1349C>T (p.Thr450Met), and c.1922C>T(p.Ser641Leu). Furthermore, AlphaMissense categorized six VUS SNVs as LP and eight as likely benign (Figure 1, Table S4). While AlphaMissense scores tend to be concordant with the ACMG evaluation, discrepancies in pathogenicity prediction occurred for variants located across distinct OCA2 functional domains.

### 3.3 Additional Pigmentation Gene Variants

We have documented additional rare variants in genes involved in pigmentation‐related pathways that may modify an OCA patient's clinical presentation. Rare variants were identified in syndromic albinism genes (*AP3B1*, *HPS6*, and *LYST*), pheomelanin regulator gene *MFSD12*, and additional OCA genes (*TYR*, *SLC45A2*, and *TYRP1*). Also annotated are observed *MC1R* variants as several individuals were described to have had red hair at birth (Table S2).

### 3.4 Structural Variation at the *OCA2* Locus

**FIGURE 3:**
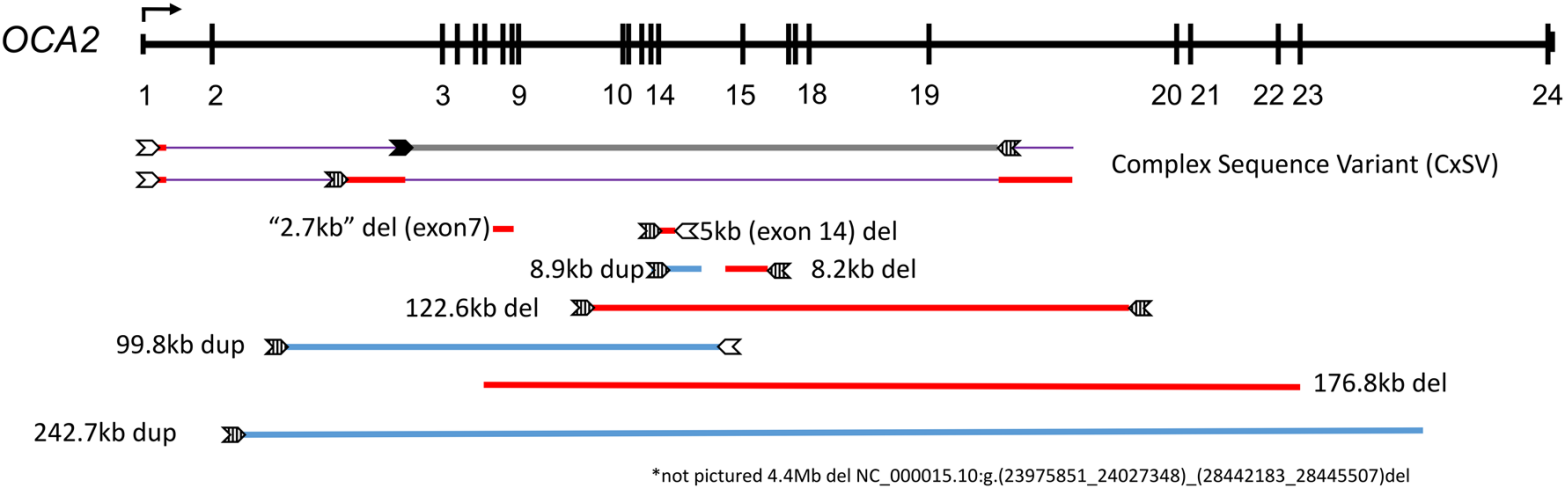
Locations of cohort structural variants in *OCA2* in relation to LINEs, SINEs, and LTR elements. Structural variants correspond to deletions (red), duplications (blue), and inversions (gray). Junctions for structural variants that reside in repetitive sequence elements are depicted as arrows. LTR elements are filled arrows, LINEs are dashed, and SINEs are not filled.

The *OCA2* locus has been well documented as prone to structural rearrangements (Rooryck et al. 2011). Eleven distinct large SVs were identified in our cohort using IGV analysis of high‐depth CCS. For eight of the SVs, either one or both junction fragment end points overlap with LINE, LTR, or SINE repetitive sequences (Figure 3). In total, 41/106 (39%) probands possessed at least one structural variant. The most frequently observed alleles were the well‐characterized exon 7 deletion (*n* = 29), the CxSV deletion variants (*n* = 8), and a 5 kb deletion spanning exon 14 (*n* = 7). GnomAD global found the prevalence of the exon 7 and exon 14 deletion alleles to be MAF = 0.0011 and MAF = 0.000046, respectively. While this exon 14 deletion allele was previously documented in a single individual (Morice‐Picard et al. 2014), we observed it in our cohort 7 times in 6 probands with self‐reported Kenyan ethnicity.

### 3.5 Splicing

Twelve rare intronic variants and one coding variant located near an exon‐intron junction (c.1636G>A; p.Glu546Lys) were evaluated for impact on splicing using a combination of splice prediction programs (Table S5). In our review of the literature, nine of these variants had been previously documented in OCA probands (Table S4). Among the four remaining variants, two reside within the canonical splice consensus (c.228‐2A>G and c.1365‐1G>C), and two reside at the +5 position (c.1116+5G>A and c.1239+5G>C). We evaluated the predicted impact on splicing for variants located outside of the canonical splice consensus relative to common frequency variant controls; all were predicted to have a deleterious effect on 5′SS junctions (Table S5).

### 3.6 Multi‐Variant Cis Haplotype Alleles

**FIGURE 4:**
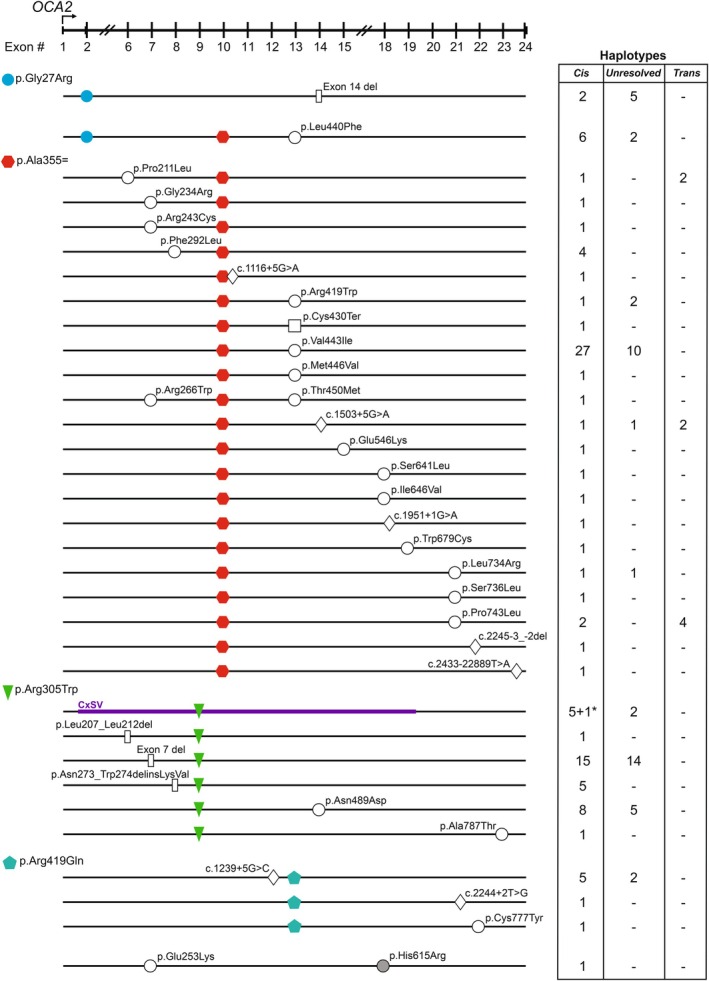
Reoccurring *OCA2* cis‐haplotypes in patient cohort. Summary of variant locations identified on distinct *OCA2* haplotypes (not to scale). Haplotype‐defining variants are filled objects. Rare missense (circles), deletion (rectangles), termination (squares), and splicing (diamonds) *OCA2* variants are open shapes. “Exon 14 del” refers to NC_000015.10: g.27979571_27984604del; “exon 7 del” refers to NC_000015.10: g.28017719_28020678delinsTTT. Phase‐validated, genotype‐consistent, and inconsistent observations of each haplotype in our cohort are listed to the right. CxSV, depicting two rare large structural variants encompassing multiple exons, is colored purple. p.Arg305Trp was identified on a cis‐haplotype 5 times with the OCA2 CxSV 143 kb inverted duplication/184 kb deletion variant (NC_000015.10:g.27874777_28094256delins[28058366_28091875;CCTGGTTGTAGGTCTAACCTGGTTAGAATCT;27898079_28040821inv;G]), and it was identified once on a haplotype with the OCA2 CxSV 143 kb inverted duplication variant (NC_000015.10:g.28091876_28094256delins[CCTGGTTGTAGGTCTAACCTGGTTAGAATCT;27898079_28040821inv;G]), which is not pictured but denoted by * in the table. Variants on a cis‐haplotype with p.Ala355= resulting in a terminated protein prior to p.Ala355= (p.Ala55Leufs*47, p.Ser92fs*10, p.Trp134Ter, p.Tyr138Ter, p.Trp204Ter, and p.Gln310Ter, respectively) were excluded from the figure.

Close evaluation of variant segregation patterns suggested that distinct multi‐variant haplotypes, comprised of rare and common frequency variants, were enriched in our cohort. We observed that 95/106 individuals with two rare variant alleles possessed additional variants that were either (1) GWAS‐identified to be correlated with pigmentation and ocular traits or (2) enriched in our cohort relative to gnomAD controls (Table S6). To resolve haplotypes for these multivariant alleles, all variants were evaluated for cis/trans orientation by paired‐end read direct sequence when possible or through segregation in informative family trios (Table S2). With respect to the alleles p.Gly27Arg, p.Arg305Trp, p.Ala355=, p.Arg419Gln, and p.His615Arg, this analysis resolved 40 phase‐validated multi‐allele haplotypes of which 10 were confirmed in multiple probands (Figure 4). For those in which phase could be resolved, 37/40 rare alleles were found in cis‐orientation only to one of these alleles, and 3 rare alleles were present in both cis and trans orientations in separate individuals with p.Ala355=.

### 3.7 c.79G>A (p.Gly27Arg) Rare Haplotypes

Review of variant allele frequencies found the variant c.79G>A (p.Gly27Arg) to be the most overrepresented allele in this cohort with an allele frequency 61‐fold greater than gnomAD allele frequencies (Table S6). This allele was observed in cis‐orientation with two distinct multi‐variant haplotypes and never on a haplotype defined by itself among OCA presenting individuals. The p.Gly27Arg and exon 14 deletion haplotype was phase‐defined by a proband with an additional 5 unphased genotype‐consistent probands identified. A separate haplotype containing p.[Gly27Arg; Ala355=; Leu440Phe] was identified in five individuals with phase‐validated haplotypes. Thus, the prevalence of the p.[Gly27Arg; Ala355=; Leu440Phe] and p.Gly27Arg and exon 14 deletion haplotypes accounts for the increased allele frequency of p.Gly27Arg in this cohort.

### 3.8 c.1065G>A (p.Ala355=) Rare Haplotypes

We document 28 distinct haplotypes in which one or more rare missense variants (*n* = 16) or predicted splice impacting variants (*n* = 5) are in cis with GWAS variant c.1065G>A (p.Ala355=) [rs1800404]. Haplotypes with this allele are of acute interest since the allele has been correlated with aberrant exon 10 splicing and thus has the potential to modulate overall *OCA2* levels (Crawford et al. 2017; Mercier et al. 2025). The most frequently identified multi‐allele rs1800404‐A containing haplotype was *OCA2* p.[Ala355=;Val443Ile] (*n* = 27). This was followed by two haplotypes identified multiple times in this dataset p.[Gly27Arg;Ala355=;Leu440Phe] (*n* = 7) and p.[Phe292Leu;Ala355=] (*n* = 4) (Figure 4, Table S2). Four of 16 missense VUS alleles residing on an rs1800404‐containing haplotype are located in the Golgi Dynamics (GOLD)‐like domain.

**FIGURE 5:**
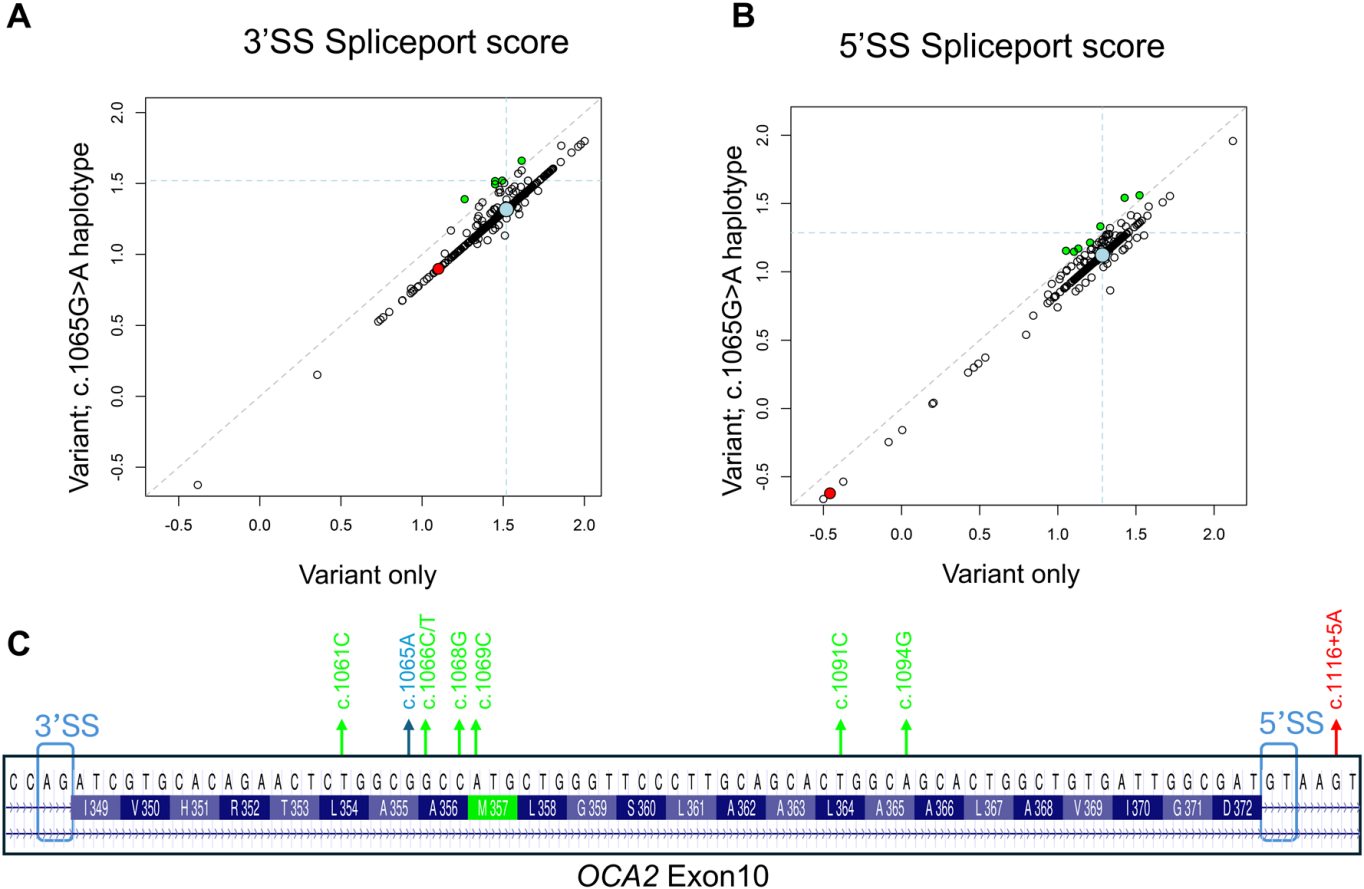
The influence of the c.1065G>A haplotype on variant impact on exon 10 splice site strength. The strength of 3′SS (A) and 5′SS (B) was quantified with SplicePort in the presence of 477 simulated individual variants (*x*‐axis) and for the same variants in cis with the c.1065G>A mutation. The variants were obtained by mutating each of the 80 positions (with the exception of the c.1065G>A position) flanking the consensus splice site dinucleotide to all possible three alternative alleles. The vertical and horizontal dashed lines correspond to the reference score of the SS (1.5194 for the 3′SS and 1.28537 for the 5′SS in figure A and B, respectively) and allow for easy assessment of variant impact on splice site strength relative to the reference sequence—decreased scores are generally interpreted as resulting in splicing aberrations (e.g., exon skipping, alternative splice sites), whereas increased scores can be interpreted as leading to higher rates of exon 10 retention. The majority of variants are located below the diagonal dashed line representing cases where the c.1065G>A haplotype decreases the strength of the splice site relative to the simulated variants alone. Five or six cases where the variant;c.1065G>A containing haplotype lead to higher scores are located above the diagonal (shown in green). The c.1116+5A variant is highlighted in red. The larger blue dot corresponds to the 3′ or 5′SS score with the reference sequence (*x*‐axis) relative to the c.1065G>A variant (*y*‐axis). (C) Exon 10 variant schematic noting the location of color‐coded variants presented in panels A and B. 3′SS and 5′SS consensus dinucleotides are noted in black boxes.

Of the five predicted splice variants in cis with rs1800404, only VUS c.1116+5G>A was located within 80 bp of rs1800404. Given their proximity, we modeled the potential impact of the two variants on each other using SplicePort. This analysis predicted c.[1065A;1116+5A] to have a more severe impact on the 5′SS than c.1116+5A alone. We next calculated all possible SplicePort values for exon 10 3′SS and 5′SS for all possible simulated haplotype variants (477) in cis with the rs1800404 alleles c.1065G vs. c.1065A (Figure 5). SplicePort scores decreased for the majority of c.1065A containing haplotypes, impacting both 3′SS and 5′SS analyses. Only 7/477 and 5/477 of these simulated cis‐variant haplotypes around the exon 10 5′SS and 3′SS, respectively, are consistent with increased exon retention and proper splicing.

### 3.9 c.913C>T (p.Arg305Trp) Rare Haplotypes

Seven distinct rare haplotypes within our cohort include the GWAS missense variant rs1800401, c.913C>T (p.Arg305Trp), (MAF = 0.05792). Three *OCA2* null‐functioning variants, the exon 7 deletion allele, 143 kb inv. dup CxSV, and 143 kb inv. dup/184 kb del CxSV, were confirmed to reside in cis‐orientation with rs1800401 (*n* = 15, *n* = 1, and *n* = 5 alleles, respectively). Rs1800401 also resides on a recurring haplotype with in‐frame deletion–insertion variants p.Asn273_Trp274delinsLysVal (*n* = 5) and p.Leu207_Leu212del (*n* = 1). Finally, rs1800401 resides on phase determined haplotypes with variants p.Asn489Asp (*n* = 8) and p.Ala787Thr (*n* = 1) (Table 1, Table S2).

### 3.10 c.1256G>A (p.Arg419Gln) and c.1844A>G (p.His615Arg) Rare Haplotypes

The common GWAS *OCA2* variant c.1256G>A (p.Arg419Gln) [rs1800407] (MAF = 0.068) has been identified in over 40 GWAS SNP:trait associations, including those for eye, skin, and hair color, retinal thickness, and skin cancer related metrics (Cerezo et al. 2025; Julian et al. 2025). Among our probands, rs1800407 was identified on haplotypes with predicted splicing variant c.1239+5G>C (*n* = 5), c.2244+2T>G (*n* = 1), and c.2330G>A (p.Cys777Tyr) (*n* = 1) (Table 1, Table S2). Though uncommon in most global populations, the frequency of c.1844A>G (p.His615Arg) [rs1800414] exceeds 50% in most of East and Southeast Asia (Kidd et al. 2020). In our cohort, proband 2431, who self‐reported Hmong ethnicity, carries p.[Glu253Lys;His615Arg] in trans with p.[Arg243Cys;Ala355=]. Additionally, proband 2431 is homozygous for a VUS duplication variant in *TYRP1* that overlaps both intron 6 and exon 7 (*TYRP1* c.1262‐1_1264dup). SpliceAI analysis of this variant produced an Acceptor Gain score of 0.99, suggesting a potential pathogenic effect. Further functional evaluation of these variants individually and the *OCA2* haplotypes would be required to determine the degree to which each allele and gene contributes to the clinical presentation.

### 3.11 Impact of rs12913832A>G on *OCA2* Expression

To quantify the degree to which rs12913832 is correlated with *OCA2* allelic expression in melanocytes, we queried high depth RNA‐Seq and genotype data from primary melanocytes previously described by Zhang et al. (2018). These data find rs12913832 the top‐ranking eQTL correlated with *OCA2* expression levels. Evaluation of genotype correlated TPM values found a 27% reduction in mean *OCA2* expression for each rs12913832‐G “blue eye allele” an individual possesses (Figure S1). These values are consistent with levels of OCA2 observed for the same genotype groupings (Cook et al. 2009) and indicate that rs12913832‐G alleles are correlated with reduced OCA2 expression levels.

Phasing of rs12913832‐G was performed with respect to the haplotypes in our cohort (Table S2). In total, 19 distinct rare haplotypes were identified in cis with rs12913832‐G. Most notably, rs1800404‐A was present in cis for 18/19 rs12913832‐G containing haplotypes.

### 3.12 Population‐Based Multi‐Variant Haplotype Frequencies

To evaluate the population specific haplotype frequency (HF) of multi‐variant *OCA2* haplotypes in this OCA2 cohort, we queried 1000 Genomes Project high‐coverage phase data and identified 10 *OCA2* cohort alleles present in the dataset. These 10 alleles were identified on 19 distinct haplotypes (Table S7). Consistent with what was found among *OCA2* cohort probands was the haplotype p.[Arg243Cys;Ala355=] occurring at a HF of 0.08%. Other rare variants were identified on distinct haplotype backgrounds. For example, p.Arg419Trp and p.Pro211Ala each individually were found only on a reference allele haplotype, HF = 0.08% and HF = 0.04%, respectively. Variant allele p.Val443Ile was detected on one of two haplotypes: p.[Ala355=;Val443Ile] (HF = 0.06%) or rs12913832‐G;p.Ala355=;p.Val443Ile (HF = 0.02%). This is consistent with what was observed among individuals carrying p.[Ala355=;Val443Ile] in our cohort.

Interestingly, several haplotypes containing only multiple common variant alleles were present. The third most frequent haplotype (HF = 14%) was comprised of common variants rs12913832‐G and p.Ala355= in cis. In addition, rs12913832‐G was found on rare haplotypes individually with common alleles p.Arg305Trp and p.Arg419Gln, with HFs of 1% and 0.4%, respectively. Also present were three‐allele haplotypes rs12913832‐G; p.Ala355=; p.Arg419Gln (HF = 0.06%) and rs12913832‐G; p.Arg305Trp; p.Ala355= (HF = 0.04%). Taken together, this analysis identified several notable multi‐allele haplotypes comprised of pigmentation trait‐associated GWAS variants and rare OCA2 variants across the *OCA2‐HERC2* locus. These haplotypes are predicted to impact protein function, gene expression, and splicing efficiency and have been identified among individuals with OCA.

## 4 Discussion

We applied the ACMG criteria to *OCA2* variants identified in a convenience cohort constructed from prior OCA studies. During this process, we were reminded that the current ACMG criteria do not include any accommodations or procedural mechanisms for assessing the pathogenicity of multiple variants contained on a single haplotype. For exceedingly rare recessive alleles, not present in large families or segregating in isolated populations, classification of single variants as pathogenic or likely pathogenic can be challenging in isolation. Evidence to support pathogenicity is frequently compiled from multiple computation tools, frequently with a “black box” type output. For a subset of *OCA2* variants, we found AlphaMissense to have provided different or incomplete pathogenicity scores in comparison to the scores obtained by applying the combined genetic, computational, and functional variant assessment approaches that comprise the ACMG framework. These discrepancies highlight the complex nature of individual variant pathogenicity assessment, which is further complicated when considering the impact of multi‐allele haplotypes.

This research demonstrates the extent to which *OCA2* rare variants are present within the context of common frequency, pigmentation trait‐associated *OCA2* alleles. Most notable are common variants rs1800404‐A and rs12913832‐G, as they each effectively decrease by 20% overall *OCA2* expression. In the case that both are present on a given non‐null allele haplotype, pathogenicity assessment would have to consider the reduction in full length *OCA2* transcript (~64% effective expression). The remaining full‐length transcript produced would be subjected to additional functional consequences of the rare variant. While we did not observe an enrichment of these alleles in our cohort, enrichment for these alleles was not anticipated given the high frequency with which they are observed in the general population. Furthermore, enrichment would only be expected if these alleles were predicted to be causative of albinism on their own, which is not the case. However, given these alleles have been associated with quantifiable albinism‐associated traits in the general population, we hypothesize that they have the potential to impact the clinical presentation associated with non‐null alleles when in cis with that allele. Thus, we would classify these loci as potential genetic modifiers of *OCA2* effective expression. For instances when these alleles are present in trans to non‐null rare alleles, no impact on phenotypic presentation attributed to that trans variant is hypothesized. Given the rare nature of these alleles, large ocular‐phenotyped cohorts will be needed to resolve the degree to which these common alleles may contribute to the phenotypic spectrum observed in albinism.

To analyze the potential implications of rs1800404‐A on *OCA2* splicing in the context of patient haplotypes, we took an in silico approach that examined multi‐variant haplotypes instead of individual rare variants relative to a single reference sequence. This approach provides a model for large scale screening of all possible patient‐specific haplotype sequences for splicing alteration implications and can be amended to analyze any gene. Synonymous variants have the potential to impact gene splicing (Gotea et al. 2015), and multiple rare variants in exon 10 have been evaluated by mini gene assays for aberrant splicing on a haplotype background containing the synonymous variant rs1800404‐A (Diallo et al. 2024; Mercier et al. 2025). For most of the rare variant rs1800404‐A haplotypes tested, an increase in improper exon 10 splicing was observed. However, Mercier et al. reported that haplotype c.[1065A;1095T] improved exon 10 retention. Our in silico analysis is consistent with these experimental findings, with most variants predicting increased aberrant splicing in the presence of rs1800404‐A except for a select few that are predicted to increase exon 10 retention. The multivariant haplotype c.[1065A;1095T] was identified as one of the 7 alleles predicted to increase proper splicing and exon 10 retention. Future functional analysis is required to better understand the process by which *OCA2* exon 10 splicing occurs, the impact of these haplotypes on OCA2 expression levels, and OCA type 2 clinical presentations.

Among individuals with OCA type 2, increasing pigmentation with age has been repeatedly described. Interestingly, this phenomenon has also been observed among individuals with homozygous *OCA2* loss‐of‐function variants and is consistent with observations for other proteins modulating conditions needed for melanin production. Overall genetic background variation has been found to impact melanin levels in *OCA2* animal models (Ishikawa et al. 2015; Lehman et al. 2000), the human *OCA2* phenotypic spectrum (King et al. 2003), and melanoma susceptibility (Baron et al. 2014; Duffy et al. 2004). This suggests that genetic background may play a role in phenotypic presentation. While infrequent, we have found 12 rare variants in pigmentation genes *TYR, TYRP1, SLC45A2, LYST, AP3B1, HPS6*, and *MFSD12*. Further investigation is needed to fully understand the contribution of multigenic variants in the context of OCA clinical severity.

We have evaluated *OCA2* rare variants in the context of in silico OCA2 protein folding predictions and the hypothesized OCA2 homodimerization (Mercier et al. 2025; Mesdaghi et al. 2023). The original in silico analyses led to the identification of an elevator structure within the inner channel of OCA2 and a luminal facing GOLD‐like domain. GOLD domains mediate protein–protein interactions and are frequently found associated with sterol‐binding domains (Mendes and Costa‐Filho 2022). Three evolutionarily conserved consensus N‐linked glycosylation sites are contained within the OCA2 GOLD‐like domain and are inferred to be important for OCA2 trafficking from the ER to the Golgi before its localization at the melanosome (Mesdaghi et al. 2023; Pastor‐Cantizano et al. 2017). Our cohort contains seven VUS located within the GOLD‐like domain. One VUS, OCA2 p.Asn273_Trp274delinsLysVal, disrupts a consensus glycosylation site within the GOLD‐like domain. Of note, this in‐frame deletion in OCA patients resides in cis on a haplotype with the common variant rs1800401‐T, which itself is located within the GOLD‐like domain. Further analysis is needed to address the role of the GOLD‐like domain in OCA2 function and how multi‐variant haplotypes in the GOLD‐like domain impact OCA2 function.

While present in the GOLD‐like domain, the functional consequence of p.Arg305Trp in isolation is unclear. We and others have observed an enriched allele frequency for p.Arg305Trp in *OCA2* patient cohorts (Branicki et al. 2008), in opposition to what would be anticipated for an albinism phenotype‐modulating allele. However, this finding has not been replicated in any of the larger OCA trait related GWAS studies. Evaluation of p.Arg305Trp by patch clamp in MNT‐1 melanoma cells finds that p.Arg305Trp does not negatively impact OCA2 function (Yang et al. 2025). For a subset of c.913C>T (p.Arg305Trp) containing chromosomes, this allele segregates in cis‐orientation with predicted null alleles. In a null allele context, it would be presumed that the p.Arg305Trp variant itself would not contribute to an OCA type 2 clinical presentation. However, these same p.Arg305Trp‐null variant haplotypes would be critical to catalog when evaluating melanoma susceptibility traits.

Also emerging from our systematic evaluation of haplotype structures was the ability to untangle contradictory literature related to the c.79G>A (p.Gly27Arg) allele. Early publications deemed p.Gly27Arg pathogenic by virtue of its low allele frequency in databases derived predominantly from European‐based populations (Garrison et al. 2004; Hutton and Spritz 2008; Oetting et al. 2005; Simeonov et al. 2013; Spritz et al. 1997). However, following the expansion of sequencing data beyond European populations, an increased MAF and homozygous (*n* = 13) unaffected individuals in African and African‐American populations were observed. We find the p.Gly27Arg allele to have a strikingly higher allele frequency (61%) than anticipated within our *OCA2* cohort. This high frequency can be attributed, in part, to the prevalence of the *OCA2* p.Gly27Arg; exon 14 deletion haplotype. Since Sanger sequencing was the standard at the time of these older publications, it is possible that the exon 14 deletion went undetected in probands who carried p.Gly27Arg. Interestingly, all individuals with the *OCA2* p.Gly27Arg; exon 14 deletion cis‐haplotype self‐identified as Kenyan, suggesting this allele should be interrogated directly for individuals of Kenyan descent. With respect to the *OCA2* p.[Gly27Arg;Ala355=;Leu440Phe] haplotype, both p.Gly27Arg and p.Leu440Phe are classified as rare variants (gnomAD MAF = 0.0011 and 0.0002, respectively). GnomAD lists both variants as present in 263 Ashkenazi Jewish probands. Given the rare MAF for both alleles, and that one of the 5 probands we confirmed to have the p.[Gly27Arg;Ala355=;Leu440Phe] cis‐haplotype was self‐reported to be of Ashkenazi Jewish descent, it suggests this is a founder allele segregating among the Ashkenazi Jewish population. Furthermore, one would predict that this haplotype in vivo would have reduced expression of the full‐length transcript as a consequence of it arising on the rs1800404‐A allele. Future analysis of patient‐derived, genotype‐appropriate primary melanocytes would be needed to confirm this hypothesis.

The remaining missense common frequency variant of note is c.1256G>A (p.Arg419Gln) [rs1800407], which we observe to be found in cis with splicing variant c.1239+5G>C. The link between rs1800407 and normal variation in eye color is well documented; however, direct functional data for the variant is limited. It is one of six variants comprising the IrisPlex predictive eye color assay, where it is associated with green/hazel iris color (Branicki et al. 2008; Pietroni et al. 2014; Walsh et al. 2012), and it is noted as a modifier of phenotypes linked to rs12913832 (Fernandez et al. 2009; Sturm et al. 2008). Rs1800407 is also associated with metastatic melanoma (Fernandez et al. 2009), corneal and refractive astigmatism (Shah et al. 2018), and retinal morphology (Currant et al. 2021). Additionally, Lasseaux et al. have highlighted that 16 OCA individuals with an unresolved molecular diagnosis have been documented to possess p.Arg419Gln in trans to a pathogenic *OCA2* variant (Lasseaux et al. 2018). Intriguingly, 1000 Genomes Project provides evidence for a rare multi‐allele haplotype [rs12913832‐G; p.Ala355=; p.Arg419Gln], which is comprised entirely of common frequency variants. Given the high rate of missing genetic heritability among individuals with OCA, further functional evaluation of this rare haplotype is needed.

Taken together, our analysis highlights the complexity present at the *OCA2* locus, including single variants, a combination of rare and common multi‐variant haplotypes, and large structural rearrangements. Detection of all alleles at the *OCA2* locus and their phase relative to each other would be best evaluated using a long read, genome‐based sequencing approach or through high depth sequencing of family trios. The diversity of haplotypes at the *OCA2* locus would suggest that the impact of individual variants on *OCA2* function should not be considered solely in isolation. For clear null alleles, a haplotype‐based analysis may be inconsequential. However, for missense or incomplete splice‐altering variants, further detailed functional analysis may be required, and we suggest they be interpreted within the context of the broader haplotypes upon which they reside. Haplotypes, composed of multiple variants which individually do not influence function but in cis can influence function, will likely play an important role in understanding the effects of variants on many genetically‐influenced phenotypes including genetic based diseases. This will become critical when identifying which variants are important in determining disease risk based on whole genome sequencing.

## Author Contributions

**Meredith F. Gillis:** conceptualization, investigation, formal analysis, data curation, visualization, writing – original draft, writing – review and editing, methodology. **Madeleine R. Ames:** investigation, formal analysis, data curation, visualization, writing – original draft, writing – review and editing, methodology. **Linnea Lundh:** conceptualization, validation, formal analysis, data curation, investigation, methodology. **Valer Gotea:** investigation, conceptualization, methodology, formal analysis, visualization, writing review and editing, software. **Laura Elnitski:** conceptualization, methodology, writing review and editing. **Frank Donovan:** formal analysis, data curation. **NISC Comparative Sequencing Program:** funding acquisition, writing – review and editing, formal analysis, data curation. **Adebowale Adeyemo:** resources, conceptualization. **Charles Rotimi:** resources, conceptualization. **Brian Brooks:** resources, conceptualization, methodology, formal analysis, writing review and editing. **Wadih Zein:** conceptualization, methodology, supervision, resources. **William Gahl:** conceptualization, writing – review and editing, funding acquisition, supervision, resources. **William S. Oetting:** conceptualization, writing – review and editing, formal analysis, supervision, resources, project administration. **David R. Adams:** writing – review and editing, visualization, formal analysis, supervision, resources, project administration, funding acquisition, conceptualization, data curation. **Stacie K. Loftus:** project administration, formal analysis, data curation, supervision, visualization, writing – review and editing, methodology, conceptualization, investigation.

## Funding

This work was supported by the National Institutes of Health (Grants NHGRI:1Z1AHG000215‐18 and NISC:1ZIBHG000196).

## Conflicts of Interest

The authors declare no conflicts of interest.

## Supporting information


**Table S1:** pcmr70085‐sup‐0001‐Tables.xlsx.

**Figure S1:** pcmr70085‐sup‐0002‐FigureS1.docx.

## Data Availability

Genomic Data will be made available through the NCBI dbGaP portal accession #phs004422.v1.
