# Supplementary material for: Haplotype‐Based Analysis of OCA2 Variants in Oculocutaneous Albinism
Source: Pigment Cell Melanoma Res. 2026 Mar 29;39(3):e70085. doi: 10.1111/pcmr.70085 (PMC13033472; doi:10.1111/pcmr.70085)
Supplement: Supplementary file 2 — Figure S1: pcmr70085‐sup‐0002‐FigureS1.docx. [file PCMR-39-0-s001.docx]

Supplemental Figure 1.

**SuppFig 1. OCA2 expression is correlated with rs12913832 genotype.** RNA-seq data from 106 primary melanocytes plotted based on OCA2 21kb upstream enhancer variant rs12913832. Expression of OCA2 is reduced by roughly 27% for each rs12913832-G allele present. Pairwise p-values were obtained with Dunn's test, (**=0.0057, ****=<0.0001) post-hoc to a significant Kruskal-Wallis test <0.0001.
